# Comparison of two transarterial chemoembolization regimens in patients with unresectable hepatocellular carcinoma: raltitrexed plus oxaliplatin *versus* 5-fluorouracil plus oxaliplatin

**DOI:** 10.18632/oncotarget.16298

**Published:** 2017-03-16

**Authors:** Wei Cui, Wenzhe Fan, Qun Zhang, Jia Wen, Yonghui Huang, Jianyong Yang, Jiaping Li, Yu Wang

**Affiliations:** ^1^ Department of Interventional Oncology, the First Affiliated Hospital, Sun Yat-sen University, Guangzhou, China; ^2^ Department of Radiotherapy, the First Affiliated Hospital, Sun Yat-sen University, Guangzhou, China; ^3^ Department of Interventional Radiology, the First Affiliated Hospital, Sun Yat-sen University, Guangzhou, China

**Keywords:** liver neoplasm, transarterial chemoembolization (TACE), raltitrexed, fluorouracil, chemotherapy

## Abstract

**Aims:**

To compare the safety and efficacy of TACE using raltitrexed, oxaliplatin and epirubicin with 5-fluorouracil, oxaliplatin and epirubicin for patients with unresectable hepatocelluar carcinoma.

**Results:**

Median overall survival (OS) was 7.4 months in the raltitrexed group [95% confidence interval (CI): 5.4, 9.4) and 5.8 months in the control group (95% CI: 5.2, 6.4; *P* = 0.177). The median progression-free survival (PFS) time was significantly higher in the raltitrexed group (3.6 months, 95% CI: 2.8, 4.4) than in the control group (2.6 months, 95% CI: 2.2, 3.0; *P* = 0.038). The disease control rate (DCR) was higher in the raltitrexed group than in the control group (40% *versus* 30.4%; *P* = 0.353). The incidence of adverse events was similar between the two groups.

**Materials and Methods:**

From January 2012 to December 2014, 86 patients with unresectable HCC were treated with TACE using the combination of raltitrexed, oxaliplatin and epirubicin (raltitrexed group), and the combination of 5-fluorouracil, oxaliplatin and epirubicin (control group). The primary endpoint was OS, and the secondary endpoints were PFS, DCR and adverse events.

**Conclusions:**

Although the study did not meet its primary endpoint, raltitrexed group reach a higher PFS, which suggests that this combination regimen of TACE as alternative may confer some benefits to selected patients.

## BACKGROUND

Transarterial chemoembolization (TACE) is a widely used treatment for unresectable hepatocellular carcinoma (HCC). It involves catheterization of the tumor feeding artery, followed by injection of embolization agents and antitumor drugs through the catheter. However, embolization is not always complete, and HCC is not very sensitive to chemotherapy.

The anticancer drugs traditionally used in TACE (e.g., doxorubicin, cisplatin, and mitomycin C) have demonstrated unsatisfactory results in patients with unresectable HCC [1]. As an alternative, a recent multicenter, open-label, randomized phase III study suggests that FOLFOX4 (infusional 5-fluorouracil, leucovorin, and oxaliplatin) administered as palliative chemotherapy may benefit patients with advanced HCC owing to better progression-free survival (PFS) and response rates compared with doxorubicin [2]. Moreover, as reported by Takahiro et al., arterial infusion chemotherapy using cisplatin, 5-fluorouracil, and leucovorin is safe and effective in patients with advanced HCC [3]. Although these studies attest to the feasibility and safety of transarterial infusion using FOLFOX4, prolonged infusion is required, which increases the risk of catheter thrombosis and biliary and hepatic side effects [4]. Additionally, leucovorin is commonly used to amplify the antitumor actions of fluoropyrimidines [5], and the complexity of fluoropyrimidine administration limits the use of the transcatheter arterial route.

Raltitrexed (also known as Tomudex) is a novel thymidylate synthase inhibitor that has anticancer effects as shown in clinical and preclinical studies of colorectal, gastric, cervical, and other cancers [6-8]. Raltitrexed inhibits HepG2 proliferation by inducing G0/G1 arrest *in vitro* and hence can potentially inhibit HCC growth *in vivo* [9]. When combined with oxaliplatin HAI, it is a feasible and promising treatment for advanced colorectal cancer [4, 10]. Unlike 5-fluorouracil, raltitrexed can definitively inhibit thymidylate synthase activity in a short infusion, and because it has a better dose-response relationship than 5-fluorouracil does, is a good candidate for transarterial perfusion [4, 10]. However, few studies have examined the safety and efficacy of raltitrexed-based TACE in patients with HCC. To identify effective and convenient therapeutic chemotherapy combinations with minimal side effects, we retrospectively compared the safety and efficacy of two TACE regimens (raltitrexed, oxaliplatin, and epirubicin *versus* 5-fluorouracil, oxaliplatin, and epirubicin) in patients with unresectable HCC.

## RESULTS

### Study subjects

Between January 2012 and December 2014, 128 consecutive patients with unresectable HCCwere retrospectively observed. Among these, 18 patients, all of whom had received raltitrexed, did not meet the inclusion criteria (six with main portal vein obstruction, one with a secondary malignancy, and eleven who had received previous treatment.). Twenty-four patients who did not receive raltitrexed were also excluded ( ten with main portal vein obstruction, two with a secondary malignancy, and twelve who had received previous treatment.). As a result, 86 patients treated with TACE were eligible for our study.

The median tumor size was 7.5 cm (range: 3.0-18.7 cm). The baseline characteristics for the control (5-fluorouracil, epirubicin, and oxaliplatin) and raltitrexed (raltitrexed, epirubicin, and oxaliplatin) cohorts are summarized in Table 1; none of these characteristics differed significantly between the groups. All patients were diagnosed with hepatitis B virus (HBV)-related HCC, and most of them were male.

**Table 1:** Baseline characteristics for all recruited patients

	Raltitrexed group *N* = 40	Control group *N* = 46	*P* value
Sex			0.243
Male	35 (87.5)	44(95.7)	
Female	5 (12.5)	2 (4.3)	
Age (years)	51.6 ± 10.0	55.4 ± 9.7	0.079
HBsAg			0.877
Positive	31 (77.5)	35 (76.1)	
Negative	9 (22.5)	11 (23.9)	
AFP, ng/mL			0.582
≤ 400	18 (45.0)	18 (39.1)	
> 400	22 (55.0)	28 (60.9)	
Tumor size, cm			0.733
≤ 10	30 (75.0)	33 (71.7)	
> 10	10 (25.0)	13 (28.3)	
Tumor number			0.200
Single	6 (15.0)	3 (6.5)	
Multiple	34 (85.0)	43 (93.5)	
Portal vein tumor thrombus			0.618
Absence	16 (40.0)	16 (34.8)	
Presence	24 (60.0)	30 (65.2)	
Ascites			0.567
Absence	34 (85.0)	41 (89.1)	
Presence	6 (15.0)	5 (10.9)	
Perfmance satus			0.835
0	4 (10.0)	4 (8.7)	
1-2	36 (90.0)	42 (91.3)	
BCLC stage			0.362
Stage B (intermediate)	15 (37.5)	13 (28.3)	
Stage C (advanced)	25 (62.5)	33 (71.7)	
Child-Pugh class			0.249
A	31 (77.5)	40 (87.0)	
B	9 (22.5)	6 (13.0)	
Extrahepatic metastases			0.965
Absence	28 (70.0)	32 (69.6)	
Presence	12 (30.0)	14 (30.4)	
Histologically confirmed	5 (12.5)	6 (13.0)	0.940

Values are mean ± standard deviation (SD) or number of subjects (%).

Abbreviations: HBsAg, Hepatitis B surface antigen; AFP, Alpha-fetoprotein; ECOG, Eastern Cooperative Oncology Group; BCLC, Barcelona Clinic Liver Cancer.

### Treatment

**Figure 1:**
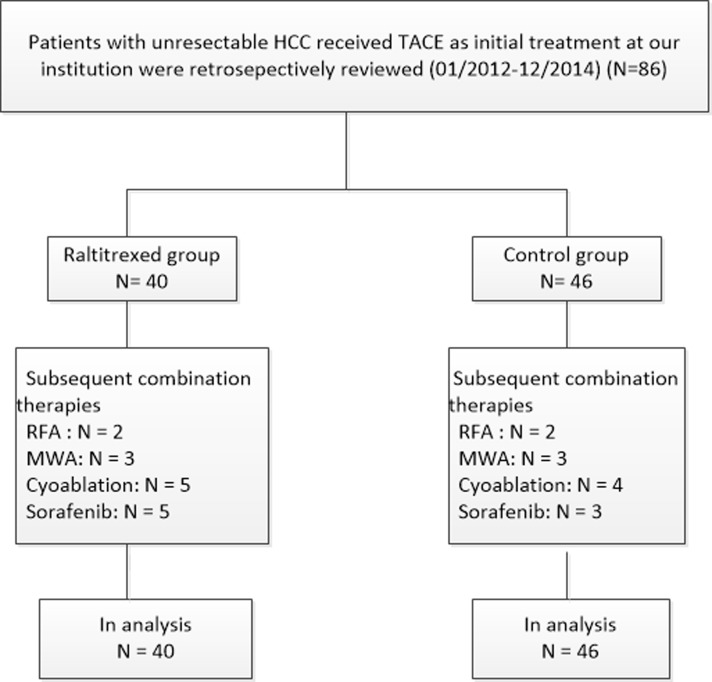
Flow diagram shows patient selection HCC, hepatocellular carcinoma; TACE, transarterial chemoembolization; RFA, radiofrequency ablation; MWA, microwave ablation.

Among the 86 patients in our study, the mean number of TACE sessions per person was 1.85 (range: 1-7 sessions, total: 159 sessions). The maximum number of TACE sessions per person in the raltitrexed and control groups were seven and six, respectively. There was no significant difference in the volume of the lipiodol-epirubicin emulsion administered in each TACE session between the raltitrexed (6.7 ± 3.4 mL) and control (7.3 ± 4.1 mL) groups (*P* = 0.476). Subsequent combination treatments included radiofrequency ablation, microwave ablation, cryoablation, and sorafenib administration. The treatments are detailed in Figure 1. There was no significant difference in the number of subsequent treatments between the raltitrexed and control groups (*P* > 0.05).

### Safety and toxicity

There were no procedure-related mortalities or 30-day mortalities. Complications are listed in Table 2. The most common complications were postembolization syndrome and liver dysfunction. Grade 3-4 adverse events occurred in five of the 40 patients in the raltitrexed group and six of the 46 patients in the control group. There was no significant difference in the grade or rate of adverse events between the two groups.

**Table 2:** Number of complications in the two groups

Incidence of complications	Raltitrexed group *N* = 40		Control group *N* = 46		P*
	Any grade	Grade 3-4	Any grade	Grade 3-4	
Postembolization					
Fever	20	2	24	1	0.841
Pain	28	0	33	0	0.859
Vomiting	13	1	17	2	0.665
Blood					
Platelets	3	1	2	1	0.533
Hemoglobin	1	0	2	0	0.476
Leukocytes	3	1	2	0	0.764
Liver function change					
Ascites/ pleura effusion	2	0	2	0	1.000
Edema: limb	1	0	2	0	1.000
Liver dysfunction	11	0	21	1	0.082
Bleeding	0	0	1	1	-

* *P* value was calculated by a two-sided χ^2^ test.

### Tumor response

Tumor response was assessed at 4 to 6 weeks, with data available for 39 of the 40 patients in the raltitrexed group and 44 of the 46 patients in the control group. The results for the two groups are shown in Table 3. The disease control rates were 40% and 30.4% for the raltitrexed and control groups, respectively, and were not significantly different (*P* = 0.353).

**Table 3:** Tumor response in two groups

Outcome	Raltitrexed group *N* = 40	Control group *N* = 46	*P* value*
Disease control rate	16	14	0.353
Complete response	0	1	
Partial response	1	1	
Stable disease	15	12	
Progressive disease	22	31	
Not evaluated†	1	2	

*P value was calculated by a two-sided χ2 test. † There were 3 patients who could not be evaluated for treatment response beacause of death, poor performance status, or patients’ refusal of evaluation at 4-6 weeks.

### Survival analysis

**Figure 2:**
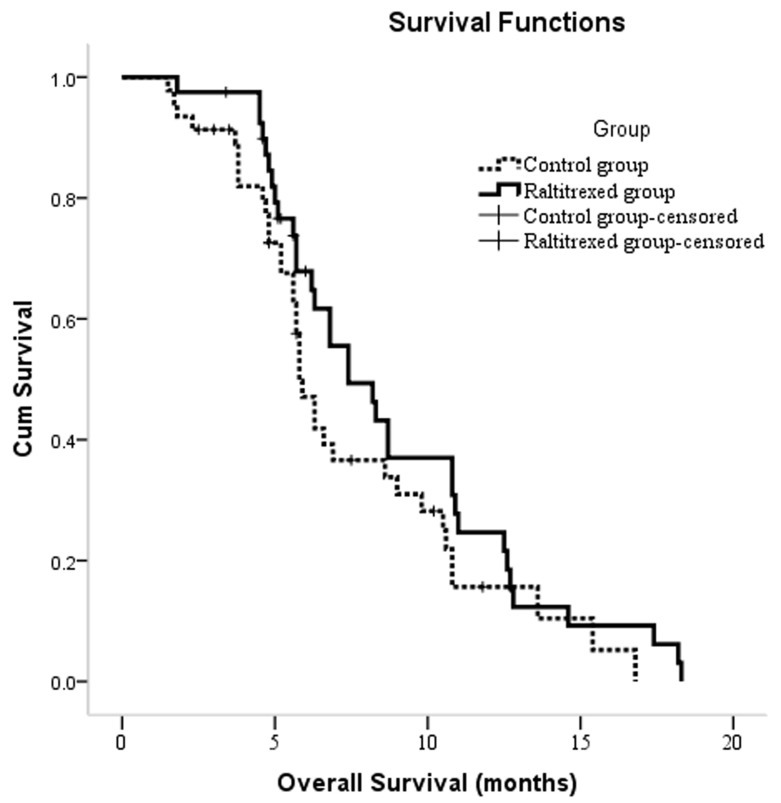
Kaplan-Meier curves of OS in patients with unresectable hepatocellular carcinoma who underwent TACE between two groups

At the end of the study period, six (15.0%) patients in the raltitrexed group and 10 (21.7%) patients in the control group were still alive. The median follow-up time was 8 months, and the total follow-up time was 18.3 months. Overall survival (OS) times for the entire cohort ranged from 1.5 to 18.3 months, with a median time of 6.6 months [95% confidence interval (CI): 5.5, 7.6]. A Kaplan-Meier survival analysis revealed no significant between-group differences in OS (*P* = 0.177), with median OS times of 7.4 (95% CI: 5.4, 9.4) and 5.8 (95% CI: 5.2, 6.4) months for the raltitrexed and control groups, respectively (Figure 2). In a univariate analysis, Barcelona Clinic Liver Cancer (BCLC) stage C (*P* < 0.001), α-fetoprotein (AFP) level ≥400 ng/mL (*P* = 0.019), tumor size ≥10 cm (P = 0.023), extrahepatic metastases (*P* = 0.002), and portal vein tumor thrombosis (*P* < 0.001) significantly correlated with poor overall survival (Table 4). In a multivariate analysis, only stage C (*P* < 0.001) was an independent predictor of poor OS.

**Table 4:** Univariate and multivariate analysis of prognostic factor for OS

Factors	No. of patients	Median OS (mo.)	*P*	Hazard ratio (95%CI)	*P*
Treatment method			0.177		
Raltitrexed group	40	7.4			
Control group	46	5.8			
Sex			0.666		
Male	79	6.3			
Female	7	8.2			
Age (years)			0.960		
≤ 60	61	6.3			
> 60	25	7.4			
ECOG Performance status			0.567		
0	8	9.8			
1-2	78	6.3			
HBsAg			0.635		
Negative	20	6.2			
Positive	66	6.8			
BCLC stage			﹤0.001	0.140 (0.069, 0.285)	﹤0.001
Stage B (intermediate)	28	12.5			
Stage C (advanced)	58	5.7			
Tumor number			0.195		
Single	9	12.7			
Multiple	77	6.6			
Child-pugh class			0.216		
A	71	6.8			
B	15	5.6			
AFP, ng/mL			0.019		0.110
≤ 400	36	8.6			
> 400	50	5.8			
Tumor size, cm			0.023		0.360
≤ 10	63	7.4			
> 10	23	5.7			
Extrahepatic metastases			0.002		0.648
Absence	60	7.4			
Presence	26	5.8			
Portal vein tumor thrombus			﹤0.001		0.380
Absence	32	10.9			
Presence	54	5.7			
Ascites			0.682		
Absence	75	6.8			
Presence	11	6.3			

Abbreviations: ECOG, Eastern Cooperative Oncology Group; HbsAg, Hepatitis B surface antigen; BCLC, Barcelona Clinic Liver Cancer; AFP, Alpha-fetoprotein.

**Figure 3:**
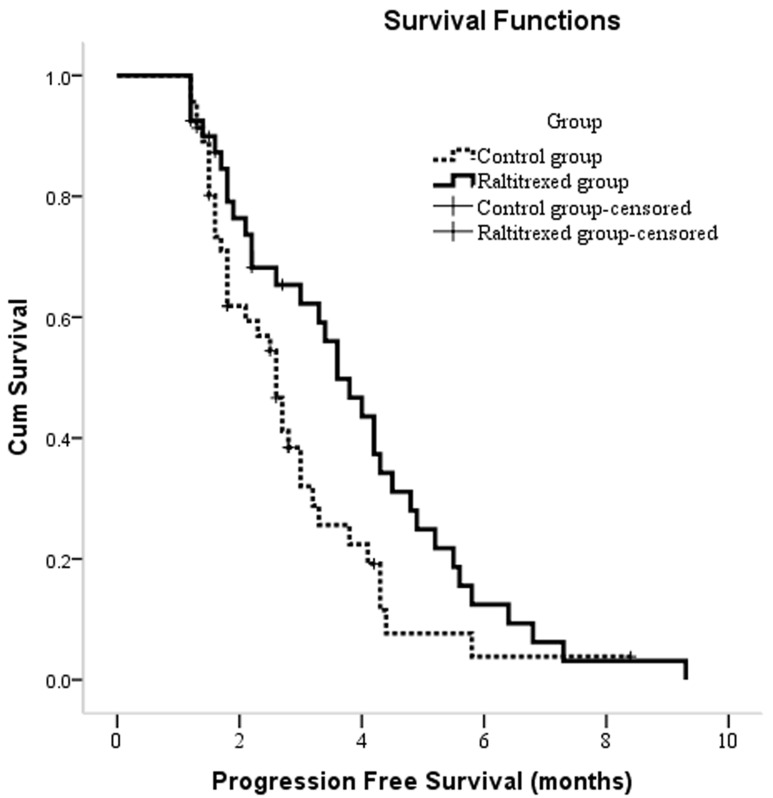
Kaplan-Meier curves of PFS in patients with unresectable hepatocellular carcinoma who underwent TACE between two groups

PFS times for the entire cohort ranged from 1.2 to 9.3 months, with a median time of 3.0 months (95% CI: 2.4, 3.7). In a Kaplan-Meier survival analysis, the median PFS time was 3.6 months (95% CI: 2.9, 4.4) in the raltitrexed group and 2.6 months (95% CI: 2.2, 3.0) in the control group, and this difference was significant (*P* = 0.038) (Figure 3).

## DISCUSSION

The FOLFOX regimen is widely used to treat gastrointestinal tumors [11, 12]. According to previous reports, it also treats unresectable HCC when administered intravenously [2, 13, 14]. Although a recent study suggests that FOLFOX4 chemotherapy is more cost-effective than is sorafenib chemotherapy for advanced HCC in China [14], the clinical results are unsatisfactory [5]. Transarterial infusion chemotherapy is a promising strategy, with a higher response rate than that of systemic chemotherapy and a positive survival trend. The effectiveness of transarterial chemotherapy mainly reflects the high local concentrations and minimal toxicity of cytotoxic drugs owing to their extraction from the hepatic arterial blood *via* the first-pass effect [3].

Transarterial infusion of FOLFOX4 is problematic because 5-fluorouracil, a component of FOLFOX4, is a time-dependent antitumor drug, and pronged perfusion increases the risk of catheter thrombosis. Although raltitrexed has no cardiac toxicity and can potentially substitute for conventional drugs such as 5-fluorouracil [15], clinical data for raltitrexed-based TACE in patients with unresectable HCC are rare. Therefore, we replaced 5-fluorouracil with raltitrexed in the FOLFOX regimen, administered *via* hepatic artery infusion, as a potential novel option for TACE.

In this retrospective study, the median OS time of all patients with unresectable HCC was 6.6 months (95% CI: 5.5, 7.6), which is similar to that reported previously [16, 17]. OS times were similar in the raltitrexed group (TACE with raltitrexed, oxaliplatin, and epirubicin) and control group (TACE with 5-fluorouracil, oxaliplatin, and epirubicin), whereas PFS times were significantly longer in the former. This result suggests that use of the raltitrexed regimen in TACE may be beneficial to patients with unresectable HCC.

Therapeutically, raltitrexed has a better *in vitro* dose-response relationship than does 5-fluorouracil and thus is a promising candidate for hepatic arterial perfusion. Raltitrexed can be administered *via* a short infusion and dramatically enhances the efficacy of oxaliplatin [10]. In a recent study of patients with unresectable HCC, raltitrexed plus oxaliplatin-based TACE had a better objective response rate than did 5-fluorouracil plus oxaliplatin- or doxorubicin plus oxaliplatin-based TACE [15]. The median OS time of raltitrexed-based TACE (13.4 months) was significantly higher than that of 5-fluorouracil-based TACE (8.5 months) and doxorubicin-based TACE (9.6 months). The results of this study show that raltitrexed-based TACE has benefits in unresectable HCC patients. Moreover, they suggest that raltitrexed may be a more suitable alternative for patients with cardiologic risk factors or previous cardiotoxicity. The survival times and tumor response rates in our study are slightly lower than those in a previous study [15], presumably because 75.8% of the HCCs in the previous study were BCLC B (intermediate) stage compared with only 32.6% of the HCCs in our study. Previous studies have shown that BCLC stage is an independent prognostic indicator in patients with unresectable HCC [18, 19], with BCLC B stage predicting a better outcome than BCLC C stage [18-20]. As confirmation, BCLC C stage was an independent risk factor for survival in the multivariate analysis in our study.

Our study also evaluated safety and tolerability of raltitrexed-based TACE in patients with unresectable HCC. We found that the incidence of major complications did not differ significantly different between the raltitrexed and control groups. All complications were reversible and adequately controlled by medical treatment. Moreover, there were no allergic reactions or cardiotoxicity during transarterial infusion with raltitrexed, and no studies to date have reported any adverse events in this context. In contrast, allergic reactions and cardiotoxicity frequently occur when antitumor drugs such as oxaliplatin and epirubicin are used [10, 21-23]. These results indicate that the combination of raltitrexed and oxaliplatin in TACE in patients with unresectable HCC is safe and tolerable.

Our study has some limitations. First, because it was retrospective, some selection bias is unavoidable. Second, because it was conducted at a single center with a relatively small number of patients, all of whom had HBV-related HCC, the results may not be generalizable to patients with HCC in Western countries. Third, the treatment protocol requires further study. In our institution and most of the hospitals in China, 5-fluorouracil is perfused for less than 48 hours. This simplifies the process of hepatic arterial infusion and avoids the risk of catheter thrombosis and biliary and hepatic side effects, but may also dampen the antitumor effects of 5-fluorouracil. Fourth, each patient received the same doses of chemotherapeutic agents, whereas doses based on body mass index may be more suitable. Fifth, TACE was performed using lipiodol and gel foam as the embolizing agents, whereas bead particles are more commonly used in Europe and the United States [24].

Although our study did not meet its primary endpoint (OS), it did show that raltitrexed-based TACE had a longer PFS time than did traditional 5-fluorouracil-based TACE and was safe and tolerable. Our findings suggest that the combination of raltitrexed, epirubicin, and oxaliplatin in TACE confers some benefits to patients with unresectable HCC. Larger prospective trials are needed to confirm this conclusion.

## MATERIALS AND METHODS

### Study design

Selected patients with unresectable HCC treated with TACE at our institution between January 2012 and December 2104 were studied. Generally, lipiodol mixed with epirubicin was slowly injected for chemoembolization, and additional antitumor drugs were injected for arterial perfusion chemotherapy. Based on their choice of chemotherapy combinations, the patients were sorted into a raltitrexed group (raltitrexed, epirubicin, and oxaliplatin) and a control group (5-fluorouracil, epirubicin, and oxaliplatin) (Figure 1). The study was approved by the Institutional Review Board of our hospital and complied with the provisions of the Declaration of Helsinki. All patients provided written informed consent.

### Study subjects

HCC was diagnosed in accordance with the guidelines of the European Association for the Study of the Liver/American Association for the Study of Liver Disease [25]. The BCLC system was used for staging [26]. Patients who met all of the following criteria were included in the analysis: (1) 18 to 75 years of age; (2) a history of chronic HBV infection; (3) Child-Pugh classification A or B; (4) Eastern Cooperative Oncology Group (ECOG) performance status 0-2; and (5) unresectable HCC, which was defined as the impossibility of completely removing the tumor or retaining a sufficient liver remnant to maintain liver function. Exclusion criteria were main portal vein obstruction, Child-Pugh C class liver function or massive ascites, severe coagulation disorders, and secondary malignancies; those that had previous treatment.

### TACE

The Seldinger technique was used to access the right femoral artery. Transcatheter arteriography was then performed to localize the tumor and obtain information about the feeding arteries. The level of transcatheter arterial infusion generally depends on the size, location, and arterial supply of the tumor. If the tumors were diffusely distributed, the level of the transcatheter arterial infusion was at the liver lobar, and the tip of the catheter was advanced into the right or left hepatic artery. If all the tumors were fed by one enlarged independent hepatic artery branch, the tip of catheter was advanced into this branch.

The patients in the raltitrexed group received a transcatheter arterial infusion containing 200 mg of oxaliplatin (Qilu Pharmaceutical Hainan Co., Ltd., Haikou, China), followed by 4 mg of raltitrexed (Nanjing Chia Tai-Tianqing Pharmaceutical Co., Ltd., Nanjing, China). The patients in the control group received a transcatheter arterial infusion containing 200 mg of oxaliplatin, followed by 1.0 g of 5-fluorouracil (Nantong Jinghua Pharmaceutical Co., Ltd., Nantong, China). To amplify the antitumor effect of 5-fluorouracil, patients in the control group received 200 mg of calcium levofolinate (Lingnan Pharmaceutical Ltd., Guangzhou, China) *via* intravenous drip 30 minutes before TACE.

For chemoembolization, a chemotherapeutic emulsion containing 20 mL lipiodol (Guerbet, Roissy, France) was mixed with 40 mg epirubicin (Farmorubicin; Pfizer, Wuxi, China). Using a 5-Fr Yashiro catheter or 2.7-Fr microcatheter (Progreat; Terumo Corp., Tokyo, Japan), the emulsion was slowly injected under digital subtraction angiography guidance until the blood flow slowed. Gel foam (350-560 μm; Ailikang Medicine Technology Co., Ltd., Hangzhou, China) or polyvinyl alcohol foam embolization particles (300, 500, or 700 μm; Cook Medical, Bloomington, IN, USA) mixed with contrast medium were used to reduce residual blood flow if necessary; they were injected until there was no longer any tumor staining after repeated angiography. The tumor-feeding artery was selected or super-selected whenever possible. Treatments were repeated every 4 to 6 weeks. The endpoints of the TACE treatment included:(1) contraindications such as main portal vein thrombosis occurred; (2) the ECOG performance status was > 2 or the Child-Pugh class was C; (3) technical difficulties prevented embolization of the residual tumors (e.g., the catheter could not reach the tumor-feeding artery); or (4) the patients refused to continue the procedure.

### Follow-up and assessment

Follow-up included routine blood tests, measurement of AFP levels, liver and kidney function, and contrast-enhanced computed tomography (CT). They occurred at 4-6 week intervals after the most recent TACE session. The criterion for retreatment was any response less than a complete response on follow-up CT imaging. If the artifacts of deposit lipidol interfered the residual tumor in CT imaging, an additonal contrast MR scan or contrast-enhanced ultrasound is usually perfomed to evaluate the residual tumor. The procedure chosen for retreatment was based on the characteristics of the recurrent and residual tumors, the patient’s preferences, and the recommendations of our multidisciplinary team.

The primary endpoint was OS, and the secondary endpoints were PFS, tumor response rate, and adverse events. The cumulative OS time was calculated from the beginning of the first TACE treatment to death or the last follow-up. PFS was defined as the time between the first TACE procedure and disease progression. The tumor response was assessed radiologically according to the modified Response Evaluation Criteria In Solid Tumors [27].

If lipiodol accumulation in the tumor was poor and showed contrast enhancement on arterial or portal venous phase contrast-enhanced CT after TACE, this was taken into account when assessing tumor burden. If the opposite was observed, the area in which lipiodol accumulated was considered necrotic, and this was taken into account when assessing reductions in tumor burden. Tumor response was classified as a complete response, a partial response, stable disease, or progressive disease. A complete response was defined as the absence of intratumoral arterial enhancement in all target lesions. A partial response was defined as a ≥30% decrease in the sum of the diameters of the viable target lesions (i.e., the lesions showing contrast enhancement in the arterial phase); the sum of the baseline diameters of the target lesions was used as the reference. Progressive disease was defined as a ≥20% increase in the sum of diameters of the viable target lesions relative to the sum at treatment initiation. Tumor responses that were neither partial nor progressive were categorized as stable disease. Adverse events were graded according to the National Cancer Institute Common Toxicity Criteria (version 3.0) [28].

### Statistical analysis

All statistical analyses were performed by using SPSS software (version 19.0; SPSS, Chicago, IL, USA). To assess the significance of intergroup differences, the continuity correction and independent samples t test, Pearson’s χ^2^ test, and Fisher’s exact test were used. Survival curves were calculated for both the raltitrexed and control groups by using the Kaplan-Meier method. Univariate analyses were performed using the log-rank test. Variables included treatment method, sex, age, ECOG performance status, hepatitis B surface antigen expression, BCLC stage, tumor number and size, Child-Pugh class, AFP level, extrahepatic metastases, portal vein tumor thrombosis, and ascites. Any variables with a *P* value < 0.10 in the univariate analysis were included in the multivariate analysis. Multivariate analyses using the Cox model were performed to identify risk factors that affected OS. *P* < 0.05 indicated a significant difference.

## Abbreviations

AFP: α-fetoprotein
BCLC: Barcelona Clinic Liver Cancer
CI: confidence interval
CT: computed tomography
ECOG: Eastern Cooperative Oncology Group
FOLFOX4: infusional 5-fluorouracil, leucovorin, and oxaliplatin
HBV: hepatitis B virus
HCC: hepatocellular carcinoma
TACE: transarterial chemoembolization
OS: overall survival
PFS: progression-free survival
